# Infrared Thermography and Physiological Variables as Methods for Recognizing Fear in Domestic Cats (*Felis catus*) Using Three Pharmacological Models: Cannabidiol, Gabapentin, and Synthetic Facial Pheromones

**DOI:** 10.3390/vetsci12060523

**Published:** 2025-05-27

**Authors:** Fabiola Torres-Bernal, Julio Martínez-Burnes, Ismael Hernández-Avalos, Adriana Olmos-Hernández, Adriana Domínguez-OIiva, Brenda Reyes-Sotelo, Cynthia González-López, Diana Villanueva-Pereyra, Daniel Mota-Rojas

**Affiliations:** 1 Science Programme “Maestria en Ciencias Agropecuarias”, Xochimilco Campus, Universidad Autónoma Metropolitana, Mexico City 04960, Mexico; 2 Facultad de Medicina Veterinaria y Zootecnia, Instituto de Ecología Aplicada, Universidad Autónoma de Tamaulipas, Victoria City 87000, Mexico; 3 Clinical Pharmacology and Veterinary Anesthesia, Biological Sciences Department, Facultad de Estudios Superiores Cuautitlán, Universidad Nacional Autónoma de México, Cuautitlán 54714, Mexico; 4 Division of Biotechnology—Bioterio and Experimental Surgery, Instituto Nacional de Rehabilitación-Luis Guillermo Ibarra Ibarra (INR-LGII), Tlalpan, Mexico City 14389, Mexico; 5 Neurophysiology, Behavior and Animal Welfare Assessment, Department of Agricultural and Animal Production, Universidad Autónoma Metropolitana (UAM), Unidad Xochimilco, Mexico City 04960, Mexico

**Keywords:** fear, cats, cannabidiol, gabapentin, synthetic facial pheromones, infrared thermography

## Abstract

This study aimed to assess changes in the surface temperature and physiological parameters of cats exposed to a fear model involving negative dog–cat interactions, receiving three pharmacological options: a single dose of cannabidiol, gabapentin, or synthetic facial pheromones. The surface temperature of the upper and lower limbs, facial, dorsal, and appendicular thermal windows was assessed through infrared thermography. Additionally, heart rate, respiratory rate, and rectal temperature were recorded. Eighty male and female domestic cats were included in the study and randomly divided into four groups: CONTROL (placebo, 2 mL/cat orally), CBD (cannabidiol, 2 mg/kg orally), GABA (gabapentin, 100 mg/cat orally), and SFP (synthetic facial pheromone, two sprays/carrier). All cats underwent six experimental phases: T_basal−_, T1st_fear_, T1st_recovery_, T_basal+_, T2nd_fear_, and T2nd_recovery_. Drug administration was carried out at the end of T1st_recovery_; the time between drug administration and T_basal+_ differed according to each drug’s nature. Statistical differences were obtained between experimental groups and times in the average surface temperature of thermal windows and cardiorespiratory parameters. In particular, the CBD, GABA, and SFP groups exhibited differences during T2nd_fear_, in contrast to the control and T1st_fear_ groups. In conclusion, the results suggest a pharmacological effect of CBD, GABA, and SFP on cats’ physiological alterations in response to fear.

## Simple Summary

In Latin America, domestic cats often experience fear during veterinary procedures; this is especially the case when several species are received in veterinary hospitals, leading to marked physiological stress responses. This study evaluated whether three pharmacological interventions—cannabidiol (CBD), gabapentin (GABA), and synthetic facial pheromones (SFPs)—could mitigate fear-induced physiological alterations in cats exposed to a negative dog–cat interaction model. Using infrared thermography, changes in surface body temperature were monitored alongside heart rate, respiratory rate, and rectal temperature. Cats receiving any of the three treatments showed a marked reduction in fear-related physiological changes compared to untreated controls. These findings suggest that CBD, GABA, and SFP can help reduce stress and improve feline welfare in fear-inducing contexts such as veterinary visits.

## 1. Introduction

The domestic cat is self-sufficient and independent, unlike conventional pets such as dogs [1,2]. However, these traits confer a greater susceptibility to negative emotional states during routine events such as transportation, veterinary visits, and introducing new members into their social group. Interspecific interactions during exploration are common in veterinary clinics of Latin America (Mexico) [3,4]. The increased sensitivity is linked to the perception of reduced environmental control, overstimulation, and perceiving their surroundings as an imminent threat [5]. This sensitivity has been reported in studies assessing physiological and behavioral responses in domestic cats exposed to recordings of multiple sounds, including dog barking, door slams, and unfamiliar voices; these are typical noises during routine veterinary procedures [6]. Likewise, the physiological and behavioral changes associated with stress are also observed during transportation [7]; however, there are limited studies regarding the impact of interspecific interactions.

Among the emotional states observed in hostile situations, fear, often accompanied by stress, is the most prevalent [8,9]. Scientific evidence suggests that fear, like other emotions, triggers a series of physiological and behavioral alterations, including organ function and animal behavior [9,10]. Specifically, during veterinary consultations and transport, fear manifests through behavioral signs such as aggression, vocalizations, evasion, and piloerection, among others, which are often considered undesirable by owners [11]. Moreover, cardiovascular and thermal modifications are also observed, which can compromise the animal’s welfare [6]. This, in turn, reduces the frequency of veterinary visits, potentially compromising the animal’s overall health and welfare [11,12]. Consequently, several pharmacological alternatives have been proposed to mitigate physiological and behavioral responses associated with fear.

One of these drugs is cannabidiol (CBD), which acts on several neuroreceptors within emotional processing centers, leading to decreased neuronal excitability and reduced perception of imminent stimuli [13]. In cats, CBD administration reduces the stress-related physiological and behavioral alterations [14]. This effect is due to the early allosteric interaction with receptors such as 5-HT, TRPV1, CB1, and GABA, among others [15,16,17], modulating emotional responses [13,18,19]. However, there is no information regarding CBD’s effect on cats’ fear responses.

Another pharmacological model with potential neuronal-transmission-blocking effects is gabapentin. Although its mechanism of action remains partially unclear, it is suggested that gabapentin inhibits the alpha-2-delta subunit of the voltage-dependent calcium channel, which is present in adrenergic, GABAergic, glutamatergic, and serotoninergic pathways [20,21,22]. Several studies have positively associated gabapentin with the modulation of stress, pain, and negative emotional states, such as anxiety, in domestic animals [23,24]. For instance, in rabbits, a single oral dose of gabapentin significantly reduced reactivity, with peak effects being observed two hours post-administration. However, it did not induce significant changes in physiological parameters such as heart rate, respiratory rate, or fecal output [23]. Similar findings have been reported in studies evaluating gabapentin’s effects on fearful feline behaviors [25,26]. However, research specifically addressing the physiological changes associated with gabapentin’s effects on fear responses in cats remains limited, highlighting the need for further investigation.

The present study also considers synthetic pheromones, which play a role in calming behaviors in domestic cats. This approach has been widely accepted by pet owners and veterinary professionals due to its practicality and ease of use, requiring no direct handling of the animal [27]. Synthetic pheromones have shown promising results in managing undesirable behaviors such as excessive scratching [28]. However, SFP has contradictory results as some studies report a minimal or absent effect during veterinary examinations [27,29]. Moreover, as with cannabidiol and gabapentin, their effects on negative emotional states, such as fear, remain poorly understood.

Currently, fear, anxiety, and pain in cats are evaluated through behavioral changes [30,31,32]. Furthermore, evidence supports the use of non-invasive tools such as infrared thermography (IRT) for evaluating animals’ emotional states [33,34]. For example, using thermal imaging, Jim et al. [35] assessed social separation stress in young cats. They found a decrease in the surface temperature of the lacrimal caruncle (a variation of 2.21 °C) following isolation. These findings are similar to those reported in cats where temperature changes were related to negative emotional states [35,36,37]. This thermoregulatory response is possibly attributed to neural mechanisms associated with fear, which activate the hypothalamic–pituitary–adrenal (HPA) axis and the sympathetic–adrenomedullary (SAM) system, leading to neurotransmitter-mediated responses via epinephrine and norepinephrine [38,39]. Domestic cats, as an endothermic species, dissipate heat from their body surface (thermal windows) into the environment, and utilize other thermoregulatory mechanisms such as salivation, panting, and behavioral adjustments [40,41]. This thermal regulation process enables infrared radiation detection through thermographic imaging, making IRT a reliable tool for emotional assessment. Notably, IRT findings have been reported to correlate with rectal temperature measurements [42].

Overall, the available literature suggests that complementary approaches, including pharmacological interventions and their assessment via IRT, represent a viable strategy. Therefore, the present study aims to identify surface microcirculatory changes using infrared thermography and cardiorespiratory parameters in response to the administration of cannabidiol, gabapentin, and synthetic facial pheromones in a fear model during a negative cat–dog interaction.

## 2. Materials and Methods

### 2.1. Study Design and Ethical Considerations

This study was a prospective, comparative experimental study. The same evaluator performed all observations and measurements. The study occurred at the private veterinary clinic in Mexico City, which is a small animal medicine facility.

A total of 80 domestic cats (*Felis catus*) were included in the study (42 males and 38 females), with an average age of 3.5 ± 1.2 years and an average body weight of 3.1 ± 1.7 kg.

Sample size calculation was based on the criteria established by Festing [43] and performed using G*Power 3.1.9.7 software [44]. The study design considered four experimental groups across six repeated measures, with an alpha error (α) of 0.05, a 95% confidence level, and a statistical power of 0.95 (1-β).

Before the experimental phase, all subjects underwent a comprehensive physical examination to rule out any clinical signs of disease or stress that could impact their performance. The evaluation included body weight, bodily secretions, posture, and behavioral repertoire assessments. Individuals exhibiting signs of acute or chronic illness (e.g., hyporexia or gingivitis), as well as those displaying abnormal behavioral patterns (e.g., aggression or excessive grooming) with the potential to interfere with study outcomes, were excluded from the study.

### 2.2. Temporary Housing Conditions Before the Inter-Species Interaction

Animals were transferred to the temporary facility room in individual carriers to allow for natural body postures. They remained confined in their carriers until the start of the experimental phase (maximum period of 30 min). This facility was located five meters from the experimental room. It was kept completely closed and secured. The temperature inside the temporary housing rooms was maintained at an average of 18–26 °C, with a relative humidity between 40 and 70%, as recommended by NOM-062-ZOO-1999.

The stimulus animal (dog) was confined and partially immobilized in a separate room from those previously mentioned. The dog was kept at a distance of 25 m to prevent pre-detection by the study subjects.

### 2.3. Treatments

**Figure 1:**
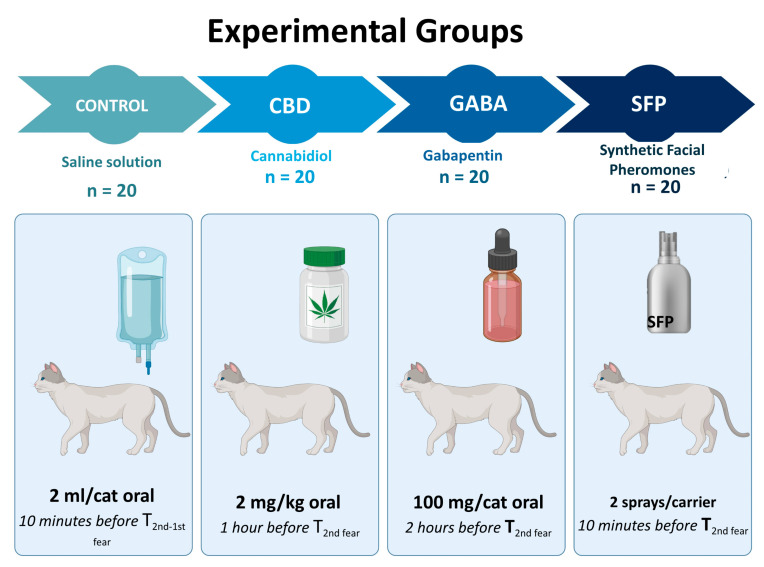
Experimental groups. Created in https://BioRender.com (accessed on 24 November 2024).

The study subjects were randomly divided using the number generation function in Microsoft Excel. Each experimental group was assigned 20 cats of indeterminate sex, classified by a pharmacological model (Figure 1):

CONTROL: A dose of 2 mL of sterile saline solution (SC Solution, PISA^®^, Toluca, Mexico) was administered orally using a syringe without a needle (Ambiderm^®^, Zapopan, Mexico), following the handling procedures outlined in NOM-062-ZOO-1999 for domestic cats.

CBD: Cannabidiol (SUPERFIELD ORGANIS^®^, Mexico City, Mexico) with a concentration of 33.3 mg/mL, was administered orally, using a 3 mL sterile syringe without needle (Ambiderm^®^, Zapopan, Mexico) at a dose of 2 mg/kg, one hour before the negative interaction (Figure 1) (according to Britch et al. [15], Rozental et al. [45], Di salvo et al. [13], Masataka [46], and previous evaluations). The individual was administered under partial immobilization, gently holding the dorsal skin of the neck and applying light pressure with the forearm to bring the individual closer to the evaluator.

GABA: A dose of 100 mg/cat of gabapentin (ALPHA CHEM^®^, Toluca, Mexico) was administered orally using a sterile 3 mL syringe (Ambiderm^®^, Zapopan, Mexico) two hours before exposure to the stimuli (Figure 1). The dose used was similar to that reported in studies by Conway et al. [23], Derek et al. [47], Di Cesare et al. [25], and Siao et al. [48], which validates the therapeutic range, among other aspects, of the drug in the species. The handling of the animals for administration was the same as in the previously described study groups.

SFP: Synthetic facial pheromone fraction F3 (FELIWAY^®^CLASSIC Spray, Loudeac, France) was administered on the corners of the inner surfaces of the carriers of each subject. Two sprays were applied 10 min before the second negative interaction protocol. The product’s technical data sheet indicates a range of +/− 8 sprays per use; thus, the dosage chosen for the current protocol follows methodologies outlined in previous studies [27,49]. No handling of the subject was required for administration due to the general design of the carriers for domestic cats.

### 2.4. Evaluation Times

**Figure 2:**
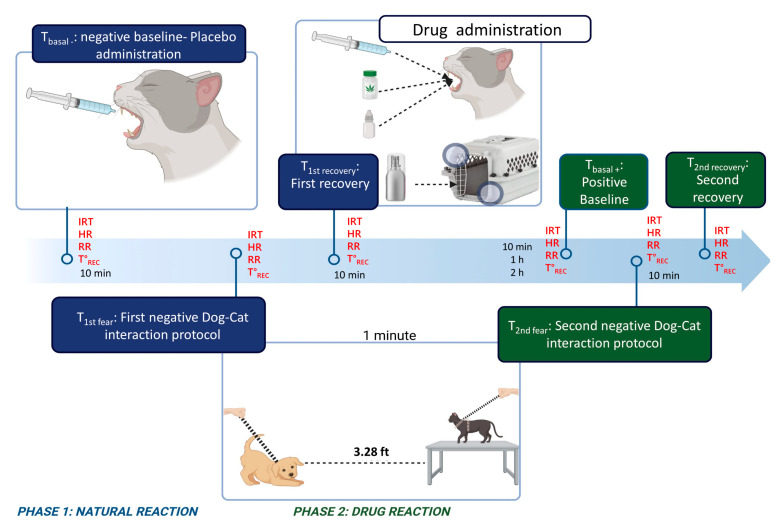
Evaluation Times. A schematic explanation of assessment stages, experimental groups, and response variables. Groups (CONTROL: negative control; CBD: cannabidiol; GABA: gabapentin; SFP: synthetic facial pheromone). Response variables (IRT: infrared thermography; HR: heart rate; RR: respiratory rate; T°_REC_: rectal temperature). Created in BioRender. https://BioRender.com/k78t117 (accessed on 24 November 2024).

All individuals went through the six experimental phases. Cats were transported in their carriers to the experimental room from T_basal−_ to the end of the experimental stage (T_1sfear_, T1st_recovery_, T_basal+_, T2nd_fear_, T2nd_recovery_). The groups were evaluated in a sequence that avoided drug interference. Thus, cats in the SFP group were the last to complete their experimental phase and waiting period. There was a 30 min interval between each group to ensure complete drug volatilization. In the negative baseline stage (T_basal−_), a placebo substance was administered 10 min before measuring the variables, except for the SFP group, where the transport box was sprayed with two atomizations of sterile, water-based placebo 10 min before the interaction. In the second stage, the cats underwent the first negative dog–cat interaction protocol (T1st_fear_) immediately after collecting the variables in the negative baseline stage. The fear protocol consisted of a forced dog–cat interaction based on the following steps: (A) The cat was removed from its transport box and placed on a table (the only object in the room covered by a non-reflective surface) at the center of the experimental room. The cat always remained on a reinforced leash and a non-reflective fabric harness. (B) The dog, with a leash and tactical harness, was introduced into the room and placed 1 m away from the table where the cat was located to ensure the safety of both individuals. (C) During the negative interaction, the dog was instructed to bark and remained in the room for one minute before being removed. The same dog was used for all treatments and events. Ten minutes after the first fear protocol, the first rest stage (T1st_recovery_) began, during which the variables were measured after recovery. After the rest period, the treatment assigned to the subject was administered. For the positive baseline stage (T_basal+_), the values were taken once the maximum concentration time for each drug had elapsed. Therefore, the time between the first rest stage and the positive baseline stage depended on the drug’s nature, which dictates its bioavailability and Tmax in the organism, as explained in Figure 1. In the fifth stage, the subjects were subjected to a second negative interaction protocol (T2nd_fear_). Finally, the rest phase (T2nd_recovery_) was repeated 10 min after the second interaction (Figure 2).

### 2.5. Assessed Parameters

#### 2.5.1. Infrared Thermography

**Figure 3:**
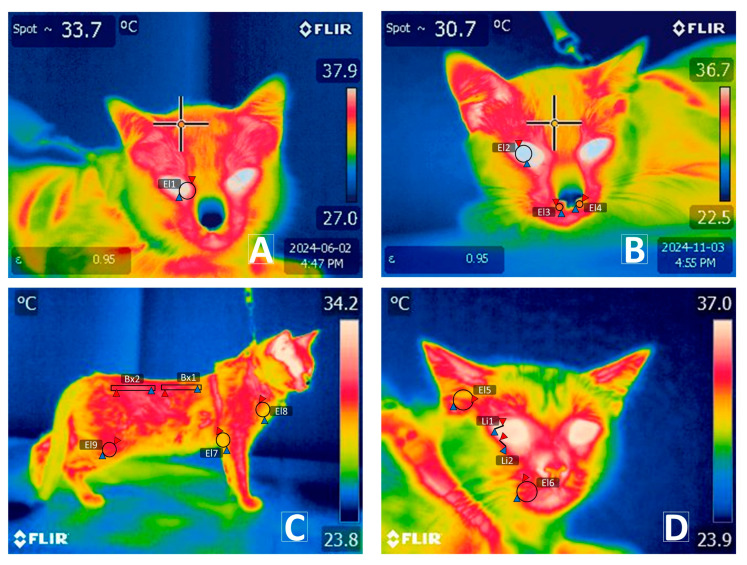
Representation of the thirteen thermal windows evaluated in domestic cats. (**A**) Thermal windows of the medial caruncle (T°_CAR_) (EI1) marked with a circular region of interest (ROI) of 2–5 mm. (**B**) Ocular window (T°_OCU_) (EI2) marked with a circular ROI of 2–5 mm. Left (T°_leftnostril_) and right nostrils (T°_rightnostril_) (EI3–EI4) delineated by 2 mm circles within the nasal openings. (**C**) Dorsal thoracic region (T°_CHEST_) (B × 1) and dorsal lumbar region (T°_lumbar_) (B × 2), outlined with a rectangular ROI of up to 10 mm in length and 2 mm in width over the upper dorsal area; thoracic limb elbow (T°_TLE_) (EI7) and thoracic limb biceps brachii (T°_TLBB_) (EI8), marked from the axillary region using a circular ROI covering a 3 mm diameter of the limb width and encompassing the vertex formed by the humeroradial joint, respectively; femoral pelvic limb (T°_FPL_) (EI9), delineated by the space along the edge of the FPL. (**D**) External auditory canal (T°_EAR_) (EI5); whiskers (T°_Whisk_) (EI6) were recorded using a circular ROI of 2 mm in diameter. Thermal windows of the lower eyelid (T°_Lowereyeline_); upper eyelid (T°_Uppereyeline_) (Li1–Li2) drawn as a line between 3 and 5 mm. Red triangles represent the highest temperatures of each ROI. Blue triangles represent the lowest temperatures of each ROI.

Thermal images were captured using an FLIR™ E80 thermal camera (FLIR Systems, Goleta, CA, USA). The camera specifications included an infrared resolution of 320 × 240 pixels, MSX resolution of 320 × 240, thermal sensitivity < 0.045 °C, accuracy of ±2 °C or ±2% at an ambient temperature of 10 °C to 35 °C, and an image frequency of 60 Hz. The emissivity value was set at 0.98, and each radiometric image was taken at an average distance of 60–80 cm with a 90° angle. Infrared representations were recorded within a fixed time frame between 08:00 and 15:00 h, with a consistent focus on the right lateral side of the cats. Non-reflective materials such as cork, rubber, kraft paper, or fabric were used to recover the table where the study subjects were positioned, to optimize thermal imaging accuracy, and minimize biased recordings. The operator wore latex gloves during the subject-handling stages to prevent thermal contamination. The operator captured two thermal images per stage, producing eight images per cat (Figure 3). The first image was focused on the rostral region, specifically targeting the anterior ocular vertex and external auditory meatus. The second image was framed to include the right lateral aspect of the cat without cropping any extremities. Thirteen thermal windows were assessed across specific anatomical regions—1–2: right lower and upper eyelids (T°_eyeline_), right lacrimal caruncle (T°_CAR_); 4: right ocular region (T°_OCU_); 5: whiskers (T°_Whisk_); 6–7: left and right nostrils (T°_nostril_); 8: right external auditory canal (T°_EAR_); 9–10: thoracic (T3–T12) (T°_CHEST_) and lumbar (L1–L7) (T°_lumbar_) vertebral regions; 11: femoral pelvic limb (T°_FPL_); 12: thoracic limb biceps brachii (T°_TLBB_); 13: thoracic limb elbow (T°_TLE_). All images were stored in JPEG format. Thermal data analysis was performed using FLIR Tools software (Version 6.4.17317.1002, FLIR Systems, Goeta, CA, USA), evaluating maximum, minimum, and average temperature ranges for each of the 13 thermal windows in the regions of interest (ROIs).

#### 2.5.2. Physiological Parameters

The measurement and recording of cardiopulmonary parameters (heart rate (HR) and respiratory rate (RR)) were obtained through direct auscultation. HR was assessed by positioning a Littmann^®^ Classic stethoscope at the 4th–5th left intercostal space in a ventral direction for one minute. RR was determined by placing the stethoscope over the bronchial pulmonary field. Rectal temperature (T°_REC_) was measured using a flexible-tip digital thermometer (Covetrus, DMT-4320, Columbus, OH, USA).

### 2.6. Procedure Description

Before the experimental phases, all cats underwent a thorough clinical examination to rule out conditions (e.g., hyporexia, fever, or gingivitis) that could interfere with subsequent assessments. Identification data were recorded, including name, age, coat pattern, density, hair length, and body condition (weight). The experimental room was prepared by installing anti-reflective materials on the table, setting up tripods and distance markers, and securing potential escape routes to ensure a controlled environment.

Environmental temperature and humidity were monitored using a hygrometer throughout the experimental phases. The same individual operated the thermal imaging camera during all sessions to maintain consistency. During resting periods and after drug administration, each cat remained inside its carrier in the holding room (a maximum period of 3 h). Upon entering the experimental room, the cat was removed from the carrier using partial restraint with a harness and leash, initiating the experimental protocol. All cats included in the study were habituated to the harness and leash.

During the negative baseline (T_basal−_), first resting period (T1st_recovery_), positive baseline (T_basal+_), and second resting period (T2nd_recovery_), physiological variables were recorded for one minute. Data were collected simultaneously with one minute of negative interaction in the fear-protocol phases (T1st_fear_ and T2nd_fear_). Positive baseline data collection (T_basal+_) began immediately after the drug’s expected onset period. Data for the first resting (T1st_recovery_) and second resting (T2nd_recovery_) phases were collected 10 min after the fear interaction stages (T1st_fear_ and T2nd_fear_), respectively.

In all phases, variable recording followed a standardized sequence: first, thermal images were taken, followed by cardiopulmonary parameters; finally, rectal temperature measurements were taken. All collected data from each subject and experimental stage were systematically recorded in a Microsoft Excel database (Microsoft Office^®^, USA).

### 2.7. Statistical Analysis

Data analysis was conducted using the statistical software package GraphPad Prism 10.4.1 (San Diego, CA, USA). The Shapiro–Wilk test was used to assess data normality. Descriptive statistics were expressed as mean ($\dot{x}$) and standard error (SE).

A repeated-measures linear mixed model was designed to evaluate the effect of the three pharmacological models (CONTROL, CBD, GABA, and SFP) across six experimental stages (T_basal−_, T1st_fear_, T1st_recovery_, T_basal+_, T2nd_fear_, and T2nd_recovery_) on the temperature differences among the thirteen thermal windows (T°_leftnostril_, T°_rightnostril_, T°_Uppereyeline_, T°_Lowereyeline_, T°_ocu_, T°_CAR_, T°_Whisk_, T°_EAR_, T°_CHEST_, T°_lumbar_, T°_TLE_, T°_FPL_, and T°_TLBB_), as well as physiological variables (HR, RR, and T°_REC_).

Multiple mean comparisons were performed using Tukey’s post-hoc test, setting the significance level at *p* < 0.05. Pearson correlation coefficients were calculated to assess the relationships between thermal windows and physiological variables.

$$Y_{ijk}=µ+\alpha_{i}+\beta_{j}+({\alpha \beta )}_{ij}+e_{ijk}$$

where:

Y_ijk_ = differences of thermal windows (T°_leftnostril_, T°_rigthnostril_, T°_Uppereyeline_, T°_Lowereyeline_, T°_OCU_, T°_CAR_, T°_Whisk_, T°_EAR_, T°_CHEST_, T°_lumbar_, T°_TLE_, T°_FPL_, and T°_TLBB_) and physiological variables (HR, RR, and T°_REC_);
µ = general mean;
α_i_ = fixed effect (CONTROL, CBD, GABA, and SFP);
*β_j_* = evaluation times (T_basal−_, T1st_fear_, T1st _recovery_, T_basal+_, T2nd_fear_, and T2nd_recovery_);
(αβ)_ij_ = interaction between treatments and evaluation times;
e_ijkl_ = random error.

### 2.8. Ethical Statement

The study was carried out in strict adherence to Mexico’s Official Standard NOM-062-ZOO-1999, which specifies the technical requirements for the humane care, management, and ethical treatment of animals involved in ethological research. The research also complied with the ARRIVE guidelines, ensuring adherence to the highest ethical principles in animal experimentation [50]. All procedures were designed to prevent harm, injury, or unnecessary discomfort for the animals, promoting their welfare throughout the study. Additionally, informed consent was obtained from the animals’ owners before initiating the procedures.

## 3. Results

Significant differences were observed in the average surface temperature of the thermal windows evaluated in domestic cats between the experimental groups and across the evaluation times. Overall, in the CBD, GABA, and SFP groups, the average surface temperature was reduced only during T1st_fear_ but remained stable during T2nd_fear_. In contrast, the CONTROL group exhibited a consistent decrease in surface temperature during the interaction periods (T1st_fear_ and T2nd_fear_). Thus, the drugs diminished the fear-related response in cats, particularly CBD and GABA treatments.

### 3.1. Upper Thermal Facial Windows

The temperature recorded at the thermal windows T°_OCU_, T°_CAR_, and T°_EAR_ decreased by at least one degree in all groups during T1st_fear_ when compared to baseline values, with the lowest recording at 32.28 ± 0.39 °C (Table 1). This reduction was also observed during T_2ndfear_ in the CONTROL group, which recorded 32.12 ± 0.33 °C as the lowest value. During T1st_recovery_, a significant temperature increase (*p* < 0.0001) was observed in all groups at the T°_CAR_ and T°_EAR_ windows, except for T°_OCU_. Meanwhile, T2nd_recovery_ showed a similar increase in the CONTROL group at T°_OCU_, T°_CAR_, and T°_EAR_.

In the case of the T°_Uppereyeline_ and T°_Lowereyeline_ windows, the surface temperature increased during T1st_fear_ in all groups compared to baseline values, with the highest recorded value being 36.18 ± 0.14 °C. The temperature consistently decreased during T1st_recovery_in all groups. In T2nd_fear_, a difference was observed only in the CBD, GABA, and SFP groups at T°_Uppereyeline_; however, the difference was generalized across all groups at the T°_Lowereyeline_.

### 3.2. Lower Thermal Facial Windows

A significant reduction in the average surface temperature (*p* < 0.0001) was recorded at T°_leftnostril_ and T°_rightnostril_ during T1st_fear_ in all groups, compared to baseline (T_basal_), with the lowest reading being 29.03 ± 0.43 °C (Table 1). This temperature significantly increased during T1st_recovery_ in all groups. Animals in the CBD, GABA, and SFP groups exhibited differences during T2nd_fear_compared to T1st_fear_. The CONTROL group maintained the reduction in temperature at the T°_leftnostril_ and T°_rightnostril_ during T2nd_fear_. Specifically, the GABA group showed differences at T_basal+._

For T°_Whisk_, a reduction in temperature was observed in the GABA and SFP groups during T2nd_fear_(33.55 ± 0.47 °C and 33.48 ± 0.42 °C, respectively), which increased during T1st_recovery_.

### 3.3. Dorsal and Appendicular Thermal Windows

T°_CHEST_had statistically significant differences between groups (*p* = 0.0002) and time points (*p* < 0.0001) (Table 1). Differences were also observed in the surface temperature of the CBD and GABA groups at T2nd_fear_(*p* = 0.0002). At T1st_fear_, all groups recorded differences when compared with T_basal−_, where the lowest value was 27.45 ± 0.27 °C, which was reestablished at T1st_recovery._

No differences were observed for T°_lumbar_, T°_TLE_, T°_FPL_, and T°_TLBB_between groups or time points.

### 3.4. Cardiorespiratory Parameters

HR and RR increased in all groups at T1st_fear_ (*p* < 0.0001) when compared with baseline values (Table 2), registering 240 ± 5.64 bpm and 93.80 ± 5.17 bpm, respectively. Similarly, the CBD, GABA, and SFP groups exhibited differences in HR at T2nd_fear_compared with T1st_fear._ In the case of RR, the CBD and GABA groups showed differences compared to CONTROL, SFP, and T1st_fear_at T2nd_fear._Similarly, differences were recorded between T1st_fear_and T1st_recovery_in all groups.

Like surface temperature, rectal temperature in the CBD, GABA, and SFP groups remained stable at T2nd_fear_ (*p* < 0.0001). In contrast, the CONTROL group showed a significant reduction (38.02 ± 0.12 °C, *p*= 0.0002) similar to T1st_fear_(Table 2). When comparing T_basal−_ vs. T1st_fear_, all groups registered a temperature decrease (*p* < 0.0001), where 37.83 ± 0.13 °C was the lowest value. At T1st_recovery_, an increase was observed, which was only repeated for the CONTROL group at T2nd_recovery_.

Positive correlations were found between thermal windows and cardiorespiratory parameters in the CONTROL (Supplementary Materials, Table S1), CBD (Supplementary Materials, Table S2), GABA (Supplementary Materials, Table S3), and SFP (Supplementary Materials, Table S4) groups, with statistically significant differences (*p* < 0.0001). Overall, a positive, strong correlation was observed between all group variables. Therefore, it is suggested that infrared thermography can be a functional tool to evaluate fear in domestic cats.

## 4. Discussion

### 4.1. Effect of the Natural Stimuli

In domestic cats, scientific findings confirm that routine procedures such as transportation, medical consultations, and even the introduction of new conspecifics into established colonies represent environmental challenges that might induce negative emotions such as anxiety and fear, which are exacerbated by the subsequent onset of stress [6,51,52,53]. Fear triggers a series of neurobiological reactions, including physiological, behavioral, and cognitive modifications to compensate for the impact of the adverse stimulus and ensure the organism’s survival [10,39,54]. In the present study, these reactions were also observed during T1st_fear_ in all experimental groups by exhibiting a significant reduction (*p* < 0.0001) in surface and rectal temperatures, in contrast to T_basal_**_−_**. Freezing is a defensive behavioral strategy to imminent stimuli such as the presence of a predator [54,55]. It is a reaction observed in cats under acute stress, and it can be observed as complete immobilization accompanied by active muscle tone, as a possible internal conditioning measure for future fight-or-flight responses [56]. This behavior agreed with the responses in the cardiopulmonary parameters of the present study. During T1st_fear_, increasing trends in HR and RR were observed in all groups compared to T_basal_, with the highest values being 240 ± 5.64 bpm (*p* < 0.0001) and 93.80 ± 5.17 bpm (*p* < 0.0001), respectively. In contrast, in murine models, freezing is observed together with bradycardia [57]. In cats, the cardiovascular changes in response to emotional state and during the fight-or-flight state can be both tachycardia and bradycardia [58,59], which is associated with the predator/prey nature of the species [60].

The received stimuli cause a neurobiological response that communicates, directly or indirectly, with the emotional processing center—the amygdala [61,62]—which sends immediate instructions to response centers such as the periaqueductal gray (PAG) [63] and the hypothalamus [64]. The neuronal population in the PAG governing the physiological and behavioral alterations leading to the freezing response [62,65]. Specifically, the ventrolateral region connects with the hypothalamic thermoregulatory center to modify the vascular diameter of the superficial capillaries, diverting a large portion of the blood volume to central circulation or regions with higher metabolic demand during the imminent stimulus [63,65]; therefore, this also suggests that the presence of the dog could deploy strategies to ensure minimal blood loss in case of significant injury. In the present study, the lowest surface temperature scores in the upper facial region were recorded at the T°_EAR_ (32.38 ± 0.29 °C) (*angularis occuli vein*) and T°_CAR_ (*Ramus auricularis intermedius*, *lateralis*, *and medialis*) (34.93 ± 0.20 °C) [66] (Figure 3). These findings are similar to those observed in rats subjected to conditioned fear contexts, in which a trend towards decreased surface temperature in the tail and paw is manifested [38].

**Figure 4:**
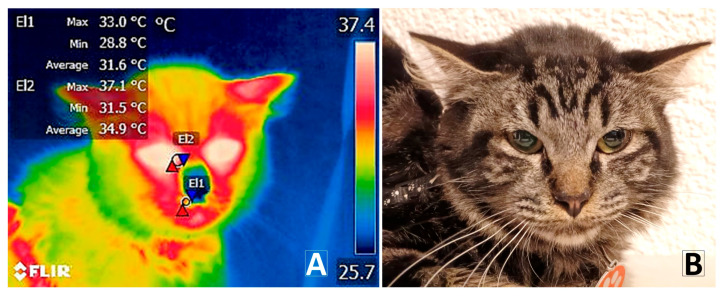
Infrared imaging in cats. (**A**) Surface temperature of the thermal windows, lacrimal caruncle (T°_CAR_) (El2), and right nostril (T°_Rightnostril_) (El1). (**B**) Digital version of the radiometric image. Red triangles represent the highest temperatures. Blue triangles represent the lowest temperatures.

On the other hand, the increase in cardiopulmonary parameters also serves as biomarkers reflecting the simultaneous manifestation of stress, involving neurotransmitters (cortisol and catecholamines) that activate the HPA and SAM axes [67]. In this regard, the alteration of cardiopulmonary patterns has also been reported in studies on domestic cats subjected to adverse challenges, such as over-stimulation by recurring noise in veterinary clinics. For example, Furgala et al. [6] observed an increase in HR and RR per minute in cats exposed to an auditory stimulus (a dog’s bark). The present study’s findings suggest strong correlations (*p* < 0.0001) between the thermal windows evaluated and the cardiopulmonary parameters. Specifically, the correlation observed between the thermal windows T°_leftnostril_, T°_rightnostril_, and RR (*p* < 0.0001) highlights the local thermal effect caused by hyperventilation (Figure 4). Like HR, breaths per minute and the rate of gas exchange are modulated by the pre-conditioning for a fight-or-flight response, and the involvement of the HPA and SAM axes [1,18]. The PAG also directly regulates the contraction of the intercostal and diaphragmatic muscles via the phrenic nerve. Furthermore, the freezing state inhibits the motor response. It exerts a form of paralysis on the abdominal muscles, making deep exhalations more difficult and shortening them until they become more frequent [68].

**Figure 5:**
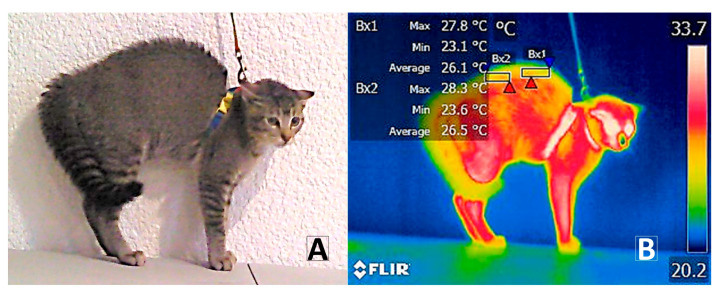
Piloerection and body posture during negative interaction. (**A**) Digital image of the piloerection response, where the increased volume is observed due to the rigid posture of the fur. (**B**) Thermographic image of the piloerection reaction. A decrease in temperature is observed in the dorsal region corresponding to the thermal windows T°_CHEST_ (B × 1) and T°_lumb_ (B × 2). Red triangles represent the highest temperatures. Blue triangles represent the lowest temperatures.

Another example of coordinated physiological responses is the significant differences (*p* < 0.0001) obtained in the T°_CHEST_window during T1st_fear_, corresponding to a peculiar physiological response in domestic animals such as cats called piloerection (Figure 5). The biological explanation lies in an increased contraction of the cardiac and skeletal muscles and the hair erector muscle (AMP) located in the dermis, causing piloerection [69,70,71]. Piloerection has been reported in multiple studies on domestic cats subjected to potential challenges such as environmental noise [6] and during the manifestation of defensive–aggressive states leading to escape [72], as well as in different species such as non-human primates under adverse aggressive stimuli [55].

On the other hand, the thermal windows T°_Uppereyeline_ and T°_Lowereyeline_ showed a significant increasing trend (*p* < 0.0001), exhibiting a maximum value of 36.38 ± 0.21 °C. In this variable, greater metabolic activity in the contraction of the *Orbicularis oculi*, *levator palpebrae*, and *retractor anguli oculi muscles* was associated, leading to increased surface temperature.

According to the present results, it is possible to suggest that monitoring the superficial temperature of thermal windows, together with the measurement of physiological parameters, represents reliable indicators in the assessment of negative emotional states, such as fear, in domestic cats [6].

### 4.2. Drug Effect

Synthetic substances [73] such as cannabidiol [45,74], gabapentin [23,24], and synthetic facial pheromones from the F3 fraction [28,49,52] have been used to reduce the intensity of emotional states such as fear in multiple species. One of the most relevant findings in the present study is the statistical difference (*p* < 0.0001) between groups during T2nd_fear,_ where CBD and GABA can be suggested as treatments due to their effect on the fear-related responses of cats. Nevertheless, the CBD, GABA, and SFP groups showed stability in surface temperatures, rectal temperature, and cardiopulmonary parameters; this is in contrast to the CONTROL group, which exhibited alterations in the study variables. Therefore, the treatments demonstrated pharmacological actions and effects that helped attenuate the physiological and thermographic responses of the cats during the negative interaction or fear response.

#### 4.2.1. Cannabidiol

The treatment that showed the most evident significant differences was CBD, as it reduced the alterations in thermal windows (T°_leftnostril_, T°_rightnostril_, T°_Uppereyeline_, T°_OCU_, T°_CAR_, T°_EAR_, and T°_CHEST_) and physiological variables (HR, RR, and T°_REC_) during T2nd_fear_. Thus, the results of the present study align with the therapeutic potential shown by cannabidiol in various studies [26,75]. Several studies have reported its potential to reduce and control fear and its anxiolytic capacity related to interaction with a wide range of receptors present throughout the organism, from cannabinoid receptors (CB1 and CB2) to receptors of serotoninergic (5-HT), GABAergic (GABA), noradrenergic (NA), and transient receptor potential vanilloid (TRPV1) systems, among others [16,18,76].

Brain mapping in cats has demonstrated a relevant number of serotoninergic (5-HT1a, 5-HT2, and 5-HT3) and NA receptors in brain regions responsible for emotional regulation, such as the amygdala, hypothalamus, and locus coeruleus (LC) [69,77]. Thus, a possible explanation for its fear control potential in cats could be that the allosteric sites of these receptors, particularly 5-HT and NA, are occupied by exogenous molecules like CBD, exhibiting a pharmacological promiscuity behavior [17]. Thus, CBD regulates the oscillations of electrical discharges and restricts the secretion of neurotransmitters such as NA, 5-HT, TRPV1, and anandamide, among others, until a state that reduces the intensity of the negative stimulus is achieved [16,78]. CBD has been shown to mitigate the intensity of fear up to extinction in rats [18], mice [79], and human models [80]; however, the present study is one of the first to report the effects of CBD in domestic cats experiencing fear.

So far, anxiolytic effects have been the most reported in cats; for example, Masataka [46] evaluated the efficacy of CBD in alleviating separation anxiety, finding a positive effect on the manifested behaviors associated with a decrease in the intensity of the negative emotion. Similarly, findings have been reported regarding relieving cardiovascular effects in models like horses [81] and dogs [82] induced by fear responses. These reports are similar to what was observed in the present study, as when contrasting the interaction times (T*1st*_fear_ vs. T*2nd*_fear_), HR remained stable in T*2nd*_fear_ (224.28 ± 6.35 bpm vs. 183.6 ± 0.20 bpm); at this point, the synthetic substance was administered. This could be due to muscle relaxation, resulting from the decline in the secretion of excitatory neurotransmitters. Similarly, excitability reduction might be associated with the stable superficial temperature values in the thermal windows during the second interaction, as the CBD group recorded 33.01 ± 0.48 °C as the minimum value, in contrast to the minimum value of the CONTROL group (29.62 ± 0.46 °C). That is, the therapeutic potential of CBD reflects a lower activity in the smooth muscle of superficial capillaries in conjunction with the accumulated effect of reduced heat production by the skeletal muscle, which diminishes thermoregulatory strategies.

Therefore, the supporting evidence suggests that the promiscuous communication of CBD promotes configurations in the neurotransmitter secretion volume, modifying neuronal communication and consequently attenuating the transmission of nerve impulses, reflecting relief in the physiological and behavioral alterations triggered by negative emotional states such as fear.

#### 4.2.2. Gabapentin

Like CBD, animals treated with gabapentin exhibited little to no alteration in superficial, rectal temperature, and cardiorespiratory parameters during T2nd_fear_, compared to the CONTROL group and the values recorded in T1st_fear_. Gabapentin administration to cats has demonstrated therapeutic potential in the treatment of pain or epilepsy [25]. Only a few studies have focused on its physiological effects on stress, anxiety, and fear responses [75,83,84,85]. For example, Pankratz et al. [26] reported a reduced exhibition of fear and stress behaviors and RR in feral cats confined in trap cages. These findings align with the present observations, in which the GABA group exhibited values of 52.85 ± 4.10 bpm during T2nd_fear_, in contrast with the CONTROL group (67.9 ± 3.56 bpm) and the average values of T1st_fear_(93.80 ± 5.17 bpm) (*p* < 0.0001). In this regard, a possible explanation for the biological function of gabapentin is its effect on the alpha-2-delta subunit of the voltage-dependent calcium channel (VGCC), reducing depolarization and cellular excitability [86,87,88]. Therefore, the evidence suggests that the generally restricted muscle dynamics dissipates progressive physiological alterations in response to fear, such as muscle contraction, vasodilation, tachycardia, and tachypnea. However, there is controversy regarding the limits of action of gabapentin or its analogs on calcium channels in specific anatomical regions or medical conditions [21].

#### 4.2.3. Synthetic Facial Pheromones

Scientific evidence published to date suggests that the efficacy of SFP is sufficient to counteract behavioral issues such as scratching in cats [28,89,90]; however, a large number of studies discuss its efficacy in potential stimuli [7,29]. Among the present findings, the SFP group exhibited reduced statistical differences compared to the GABA and CBD groups; it behaved similarly in RR during the interaction periods, although the results suggest an attenuating effect on physiological alterations in response to fear. This is in agreement with studies supporting the reduction in stress responses due to the effect of SFP in routine procedures such as transport, veterinary consultation [90], clinical handling [91], and agonistic interactions between conspecifics [92]. However, the evaluation of changes in physiological parameters due to SFP is scarce, as the focus is more on behavioral adjustments than negative emotional responses such as fear.

Neurophysiological and pharmacological foundations support the modulatory function of the synthetic portion (F3) of the facial pheromones’ synthetic portion (F3) [93]. These molecules enter the body through the nasopalatine channel to the vomeronasal organ and, subsequently, the accessory olfactory bulb [29,93,94].

#### 4.2.4. Limitations and Recommendations for Further Studies

The interaction of synthetic molecules such as CBD, GABA, and SFP modifies cellular dynamics, attenuating negative emotional states, and induces neurological adjustments. The use of these substances might alter some parameters. For example, gabapentin significantly alters the gait and body posture as a result of generalized muscle relaxation and neuronal desensitization, which might lead to inaccurate diagnoses [95]. Likewise, some studies report a high frequency of side effects such as vomiting, ataxia, and tremors after gabapentin administration, which is objectionable for owners [47,88]. These side effects were not observed in the present study.

Likewise, the pharmacological interaction of CBD, GABA, and SFP with in-clinic treatment needs further evaluation; thus, it is recommended to acknowledge the possible interactions and the recommended time to avoid adverse pharmacological effects.

In the present study, only physiological parameters were evaluated, not including behavioral manifestations, as no behavioral evaluation was performed. However, several studies have shown significant behavioral differences under the influence of CBD [82,96], GABA [73,97], and SFP [28,91,98]. Therefore, these results reflect the need for a broad approach to assessing animal emotions, considering physiological indicators, blood analytes, and behavioral patterns. Although FC, FR, and T_REC_were analyzed at the end of each experimental phase, the animals’ management was performed by unknown evaluators, which might be an additional stressor.

## 5. Conclusions

According to the values observed in each of the study groups and during the experimental stages, the findings suggest that the application of pharmacological models such as CBD, GABA, and SFP mitigate microvascular thermal alterations and cardiopulmonary modifications in fearful cats, with CBD and GABA being the models with the most satisfactory results. Therefore, their administration is recommended as support during routine procedures such as transport, veterinary consultation, and social interactions.

On the other hand, the natural physiological changes in response to fear included a significant reduction in both superficial and rectal temperature, and an increase in cardiopulmonary parameters.

Additionally, during the assessment and recognition of fear, it is recommended to employ diagnostic tools such as IRT, as the present results exhibited strong positive correlations between the physiological parameters HR, RR, and rectal temperature and the superficial temperature of the thermal windows evaluated.

## Figures and Tables

**Table 1:** Mean and standard error (SEM) of the temperatures (°C) of the 13 thermal windows of four experimental groups during six evaluation times.

Thermal Windows	Groups	T_basal−_	T1st_fear_	T1st_recovery_	T_basal+_	T2nd_fear_	T2nd_recovery_	*p*-Value
T°_OCU_	CONTROL (n = 20)	36.74 ± 0.15 ^a,1^	35.59 ± 0.19 ^b,1^	36.60 ± 0.13 ^a,1^	36.61 ± 0.09 ^a,1^	35.73 ± 0.16 ^b,1^	36.73 ± 0.15 ^a,1^	***p *= 0.0004**
	CBD (n = 20)	36.95 ± 0.19 ^a,b,1^	36.18 ± 0.18 ^a,1^	37.0 ± 0.18 ^b,1^	37.06 ± 0.17 ^a,b,1^	36.95 ± 0.14 ^a,b,2^	37.12 ± 0.16 ^b,1^	***p *= 0.0117**
	GABA (n = 20)	37.0 ± 0.18 ^a,1^	36.01 ± 0.26 ^b,1^	37.03 ± 0.22 ^a,1^	36.97 ± 0.19 ^a,b,1^	36.79 ± 0.14 ^a,b,2^	36.78 ± 0.14 ^a,b,1^	***p *= 0.0013**
	SFP (n = 20)	36.96 ± 0.20 ^a,1^	36.22 ± 0.21 ^b,1^	35.27 ± 1.86 ^a,b,1^	37.18 ± 0.14 ^a,1^	36.91 ± 0.14 ^a,b,2^	36.98 ± 0.14 ^a,b,1^	***p *= 0.0003**
*p*-Value		*p *> 0.05	*p *> 0.05	*p *> 0.05	*p *> 0.05	***p *= 0.0067**	*p *> 0.05	
T°_CAR_	CONTROL (n = 20)	36.1 ± 0.20 ^a,1^	35.18 ± 0.22 ^b,1^	36.13 ± 0.26 ^a,1^	36.33 ± 0.17 ^a,1^	35.05 ± 0.20 ^b,1^	36.64 ± 0.21 ^a,1^	***p *< 0.0001**
	CBD (n = 20)	36.62 ± 0.24 ^a,1^	35.34 ± 0.18 ^b,1^	36.92 ± 0.23 ^a,1^	37 ± 0.24 ^a,1^	36.95 ± 0.21 ^a,2^	36.88 ± 0.18 ^a,1^	***p *< 0.0001**
	GABA (n = 20)	36.89 ± 0.16 ^a,1^	34.93 ± 0.20 ^b,1^	37.12 ± 0.19 ^a,1^	36.83 ± 0.26 ^a,1^	36.58 ± 0.21 ^a,2^	36.67 ± 0.22 ^a,1^	***p *< 0.0001**
	SFP (n = 20)	36.6 ± 0.20 ^a,1^	35.51 ± 0.22 ^b,1^	36.91 ± 0.18 ^a,1^	36.87 ± 0.16 ^a,1^	36.52 ± 0.20 ^a,2^	36.74 ± 0.18 ^a,1^	***p *< 0.0001**
*p*-Value		*p* > 0.05	*p* > *0.05*	*p* > 0.05	*p* > 0.05	***p* = 0.0008**	*p* > 0.05	
T°_Uppereyeline_	CONTROL (n = 20)	35.39 ± 0.22 ^a,1^	36.18 ± 0.14 ^b,1^	35.38 ± 0.20 ^a,1^	35.17 ± 0.16 ^a,1^	35.72 ± 0.12 ^b,1^	35.30 ± 0.21 ^a,1^	***p *= 0.0004**
	CBD (n = 20)	34.85 ± 0.28 ^a,1^	36.05 ± 0.16 ^b,1^	35.29 ± 0.25 ^a,1^	35.26 ± 0.22 ^a,1^	35.12 ± 0.22 ^a,1^	35.23 ± 0.23 ^a,1^	***p *= 0.0041**
	GABA (n = 20)	35.17 ± 0.15 ^a,1^	35.88 ± 0.17 ^b,1^	34.94 ± 0.20 ^a,b,1^	34.85 ± 0.25 ^a,b,1^	34.89 ± 0.18 ^a,1^	34.93 ± 0.18 ^a,1^	***p *= 0.0237**
	SFP (n = 20)	34.76 ± 0.23 ^a,1^	35.81 ± 0.20 ^b,1^	34.76 ± 0.21 ^a,1^	34.78 ± 0.16 ^a,1^	34.76 ± 0.21 ^a,1^	34.74 ± 0.20 ^a,1^	***p *< 0.0001**
*p*-Value		*p *> 0.05	*p *> 0.05	*p *> 0.05	*p *> 0.05	*p *= 0.0672	*p *> 0.05	
T°_Lowereyeline_	CONTROL (n = 20)	35.13 ± 0.17 ^a,1^	36.05 ± 0.12 ^b,1^	35.31 ± 0.22 ^a,1^	35.28 ± 0.16 ^a,1^	35.02 ± 0.52 ^a,1^	35.06 ± 0.15 ^a,1^	***p *= 0.0004**
	CBD (n = 20)	34.92 ± 0.25 ^a,1^	36.16 ± 0.17 ^b,1^	35.44 ± 0.26 ^a,b,1^	35.31 ± 0.21 ^a,b,1^	35.03 ± 0.21^a,1^	35.23 ± 0.22 ^a,1^	***p *= 0.0002**
	GABA (n = 20)	35.39 ± 0.20 ^a,1^	36.38 ± 0.21 ^b,1^	35.39 ± 0.22 ^a,1^	35.67 ± 0.16 ^a,b,1^	35.33 ± 0.15 ^a,1^	35.51 ± 0.15 ^a,1^	***p *= 0.036**
	SFP (n = 20)	34.68 ± 0.23 ^a,1^	35.99 ± 0.18 ^b,1^	34.90 ± 0.27 ^a,1^	35.03 ± 0.16 ^a,1^	34.99 ± 0.22 ^a,1^	35.01 ± 0.19 ^a,1^	***p *< 0.0001**
*p*-Value		*p *> 0.05	*p *> 0.05	*p *> 0.05	*p *> 0.05	*p *> 0.05	*p *> 0.05	
T°_RigthNostril_	CONTROL (n = 20)	31.27 ± 0.35 ^a,1^	29.03 ± 0.43 ^b,1^	31.67 ± 0.44 ^a,1^	31.45 ± 0.41 ^a,1^	29.66 ± 0.42 ^b,1^	31.78 ± 0.40 ^a,1^	***p *< 0.0001**
	CBD (n = 20)	32.85 ± 0.56 ^a,1^	30.67 ± 0.51 ^b,1^	32.82 ± 0.69 ^a,1^	33.08 ± 0.47 ^a,1^	33.11 ± 0.51 ^a,2^	32.86 ± 0.46 ^a,1^	***p *< 0.0001**
	GABA (n = 20)	34.84 ± 0.33 ^a,1^	31.90 ± 0.62 ^b,1^	35.03 ± 0.47 ^a,1^	34.47 ± 0.39 ^a,2^	34.20 ± 0.47 ^a,2^	34.47 ± 0.45 ^a,2^	***p *< 0.0001**
	SFP (n = 20)	32.90 ± 0.51 ^a,1^	31.31 ± 0.47 ^b,1^	33.66 ± 0.46 ^a,1^	33.27 ± 0.35 ^a,1^	33.01 ± 0.48 ^a,2^	33.17 ± 0.40 ^a,1^	***p *< 0.0001**
*p*-Value		*p *> 0.05	*p *> 0.05	*p *> 0.05	***p *= 0.0007**	***p *= 0.0002**	***p *= 0.0254**	
T°_LeftNostril_	CONTROL (n = 20)	31.17 ± 0.39 ^a,1^	29.18 ± 0.47 ^b,1^	31.24 ± 0.54 ^a,1^	31.12 ± 0.45 ^a,1^	29.62 ± 0.46 ^b,1^	31.64 ± 0.45 ^a,1^	***p *< 0.0001**
	CBD (n = 20)	33.02 ± 0.51 ^a,1^	30.55 ± 0.39 ^b,1^	32.72 ± 0.63 ^a,1^	33.02 ± 0.52 ^a,1^	32.88 ± 0.49 ^a,2^	33.08 ± 0.48 ^a,1^	***p *= 0.0028**
	GABA (n = 20)	34.0 ± 0.23 ^a,1^	30.78 ± 0.47 ^b,1^	34.67 ± 0.32 ^a,1^	34.09 ± 0.29 ^a,2^	33.78 ± 0.34 ^a,2^	33.64 ± 0.32 ^a,1^	***p *= 0.0005**
	SFP (n = 20)	33.38 ± 0.47 ^a,1^	31.82 ± 0.54 ^b,1^	33.76 ± 0.48 ^a,1^	33.45 ± 0.40 ^a,1^	33.16 ± 0.45 ^a,2^	33.40 ± 0.41 ^a,1^	***p *< 0.0001**
*p*-Value		*p *> 0.05	*p *> 0.05	*p *> 0.05	***p *= 0.0343**	***p *= 0.0033**	*p *> 0.05	
T°_Whisk_	CONTROL (n = 20)	33.39 ± 0.33 ^a,1^	32.49 ± 0.33 ^a,1^	31.51 ± 0.27 ^a,1^	33.22 ± 0.27 ^a,1^	32.63 ± 0.31 ^a,1^	33.43 ± 0.24 ^a,1^	***p *= 0.081**
	CBD(n = 20)	34.27 ± 0.32 ^a,b,1^	33.48 ± 0.34 ^a,1^	34.40 ± 0.33 ^b,1^	34.44 ± 0.32 ^a,b,1^	34.30 ± 0.31 ^a,b,1^	34.39 ± 0.30 ^a,b,1^	***p *= 0.0023**
	GABA (n = 20)	34.88 ± 0.29 ^a,1^	33.55 ± 0.47 ^b,1^	35.01 ± 0.35 ^a,1^	34.55 ± 0.44 ^a,b,1^	34.30 ± 0.32 ^a,b,1^	34.48 ± 0.36 ^a,b,1^	***p *= 0.0012**
	SFP (n = 20)	34.21 ± 0.29 ^a,1^	33.48 ± 0.32 ^b,1^	34.31 ± 0.31 ^a,1^	34.19 ± 0.24 ^a,b,1^	34.05 ± 0.24 ^a,b,1^	34.09 ± 0.25 ^a,b,1^	***p *= 0.0004**
*p*-Value		*p *> 0.05	*p *> 0.05	*p *> 0.05	*p *> 0.05	*p *= 0.129	*p *> 0.05	
T°_EAR_	CONTROL (n = 20)	35.14 ± 0.29 ^a,1^	32.28 ± 0.39 ^b,1^	33.39 ± 0.34 ^a,1^	33.95 ± 0.28 ^a,1^	32.12 ± 0.33 ^b,1^	34.01 ± 0.35 ^a,1^	***p *< 0.0001**
	CBD (n = 20)	34.79 ± 0.43 ^a,1^	32.69 ± 0.28 ^b,1^	34.24 ± 0.35 ^a,1^	34.69 ± 0.39 ^a,1^	34.91 ± 0.35 ^a,2^	34.74 ± 0.35 ^a,1^	***p *< 0.0001**
	GABA (n = 20)	35.24 ± 0.27 ^a,1^	33.26 ± 0.40 ^b,1^	35.36 ± 0.31 ^a,1^	35.02 ± 0.31 ^a,1^	35.19 ± 0.22 ^a,2^	34.91 ± 0.27 ^a,1^	***p *< 0.0001**
	SFP (n = 20)	35.34 ± 0.19 ^a,1^	32.80 ± 0.34 ^b,1^	34.0 ± 0.57 ^a,1^	34.72 ± 0.31 ^a,1^	35.0 ± 0.28 ^a,2^	34.61 ± 0.33 ^a,1^	***p *< 0.0001**
*p*-Value		*p *> 0.05	*p *> 0.05	*p *> 0.05	*p *> 0.05	***p *= 0.0002**	*p *> 0.05	
T°_CHEST_	CONTROL (n = 20)	29.03 ± 0.21 ^a,1^	27.45 ± 0.27 ^b,1^	28.28 ± 0.31 ^a,b,1^	28.63 ± 0.26 ^a,1^	27.51 ± 0.28 ^b,1^	28.96 ± 0.28 ^a,1^	***p *< 0.0001**
	CBD (n = 20)	30.51 ± 0.42 ^a,1^	29.12 ± 0.41 ^b,1^	29.91 ± 0.41 ^a,1^	30.37 ± 0.38 ^a,2^	30.39 ± 0.40 ^a,2^	30.32 ± 0.39 ^a,1^	***p *= 0.0001**
	GABA (n = 20)	29.83 ± 0.34 ^a,1^	28.33 ± 0.40 ^b,1^	29.89 ± 0.41 ^a,1^	29.64 ± 0.42 ^a,1^	29.85 ± 0.39 ^a,2^	29.87 ± 0.40 ^a,1^	***p *= 0.0024**
	SFP (n = 20)	29.12 ± 0.27 ^a,b,1^	28.66 ± 0.41 ^b,1^	29.10 ± 0.39 ^a,b,1^	29.06 ± 0.36 ^a,b,1^	29.14 ± 0.39 ^a,b,1^	29.22 ± 0.38 ^a,1^	***p *= 0.0469**
*p*-Value		*p *> 0.05	*p *> 0.05	*p *> 0.05	***p *= 0.0296**	***p *= 0.0002**	*p *> 0.05	

^a,b^ Different initials indicate significant differences (*p* < 0.05) between events (T_basal__−_,T1st_fear_, T1st_recovery_, T_basal__+_,T2nd_fear_, and T2nd_recovery_). ^1,2^ Different numerals indicate significant differences (*p* < 0.05) between treatments (CONTROL,CBD, GABA, and SFP). Bold *p*-values represent statistically significant differences between events and treatments. Abbreviations of treatments: CONTROL—placebo; CBD—cannabidiol; GABA—gabapentin; SFP—synthetic facial pheromone). Abbreviations of evaluation phases: T_basal__−_—basal; T1st_fear_—first negative dog–cat interaction; T1st_recovery_—first rest; T_basal+_:—neutral after drug administration; T2nd_fear_—second negative dog–cat interaction; T2nd_recovery_—second rest. Thermal windows: T°_leftnostril_, T°_rigthnostril_, T°_Uppereyeline_, T°_Lowereyeline_, T°_OCU_ (ocular), T°_CAR_ (caruncle), T°_Whisk_ (whiskers), T°_EAR_ (external auditory canal), T°_CHEST_ (dorsal thoracic region between 3 and 12 vertebrae).

**Table 2:** Mean and standard error (SEM) of the cardiorespiratory parameters and rectal temperature of cats assigned to four experimental groups during six evaluation times.

Parameters	Groups	T_basal−_	T1st_fear_	T1st_recovery_	T_basal+_	T2nd_fear_	T2nd_recovery_	*p*-Value
Heart rate (bpm)	CONTROL (n = 20)	189 ± 8.00 ^a,1^	240 ± 5.64 ^b,1^	194.5 ± 7.73 ^a,1^	202.1 ± 6.53 ^a,b,1^	230.6 ± 6.74 ^b,1^	199.9 ± 5.06 ^a,1^	*p *< 0.0001
	CBD (n = 20)	176.1 ± 4.71 ^a,1^	222.4 ± 6.35 ^b,1^	181.4 ± 7.44 ^a,1^	177.8 ± 4.86 ^a,1^	183.6 ± 4.85 ^a,2^	179.2 ± 4.81 ^a,1^	*p *< 0.0001
	GABA (n = 20)	173 ± 6.94 ^a,1^	226.8 ± 7.95 ^b,1^	184.4 ± 9.65 ^a,1^	174.3 ± 7.35 ^a,1^	177.9 ± 7.44 ^a,2^	177.8 ± 7.07 ^a,1^	*p *< 0.0001
	SFP (n = 20)	178.4 ± 6.60 ^a,1^	229.7 ± 7.61 ^b,1^	186.8 ± 5.94 ^a,1^	183 ± 5.05 ^a,1^	185.8 ± 5.04 ^a,2^	185.7 ± 4.49 ^a,1^	*p* < 0.0001
*p*-Value		*p* > 0.05	*p* > 0.05	*p* > 0.05	*p* > 0.05	***p *= ** **0.0058**	*p* > 0.05	
Respiratory rate (bpm)	CONTROL (n = 20)	52.50 ± 3.15 ^a,1^	71.85 ± 3.89 ^b,1^	54.45 ± 4.38 ^a,1^	51.75 ± 3.08 ^a,1^	67.9 ± 3.56 ^b,1^	53.45 ± 3.73 ^a,1^	*p *< 0.0001
	CBD (n = 20)	53.15 ± 4.46 ^a,1^	81.60 ± 5.59 ^b,1^	64.95 ± 6.10 ^a,1^	51.50 ± 3.98 ^a,1^	51.20 ± 3.52 ^a,2^	47.30 ± 3.30 ^a,1^	*p *< 0.0001
	GABA (n = 20)	60.15 ± 3.95 ^a,c,1^	93.80 ± 5.17 ^b,1^	74.20 ± 5.93 ^a,c,1^	54.15 ± 4.22 ^a,1^	52.85 ± 4.10 ^a,2^	51.85 ± 4.34 ^a,1^	*p *< 0.0001
	SFP (n = 20)	59.40 ± 3.40 ^a,1^	88.40 ± 3.25 ^b,1^	64.70 ± 2.80 ^a,1^	60.15 ± 5.45 ^a,1^	62.60 ± 5.33 ^a,1^	62.60 ± 5.48 ^a,1^	*p *= 0.0004
*p*-Value		*p* > 0.05	*p* > 0.05	*p* > 0.05	*p* > 0.05	***p *= 0.0108**	*p* > 0.05	
Rectal temperature (°C)	CONTROL (n = 20)	38.69 ± 0.16 ^a,1^	37.83 ± 0.13 ^b,1^	38.29 ± 0.17 ^a,1^	38.58 ± 0.17 ^a,1^	38.02 ± 0.12 ^b,1^	38.39 ± 0.12 ^a,b,1^	*p *= 0.0003
	CBD (n = 20)	38.90 ± 0.11 ^a,1^	37.95 ± 0.12 ^b,1^	38.50 ± 0.09 ^a,1^	38.54 ± 0.11 ^a,1^	38.62 ± 0.10 ^a,2^	38.45 ± 0.10 ^a,1^	*p *< 0.0001
	GABA (n = 20)	38.93 ± 0.11 ^a,1^	37.91 ± 0.13 ^b,1^	38.62 ± 0.13 ^a,1^	38.81 ± 0.08 ^a,1^	38.84 ± 0.10 ^a,2^	38.63 ± 0.12 ^a,1^	*p *< 0.0001
	SFP (n = 20)	39.21 ± 0.09 ^a,1^	38.18 ± 0.15 ^b,1^	38.93 ± 0.10 ^a,1^	39.03 ± 0.08 ^a,1^	38.97 ± 0.10 ^a,2^	38.98 ± 0.08 ^a,1^	*p *< 0.0001
*p*-Value	*p* > 0.05	*p* > 0.05	*p* > 0.05	*p* > 0.05	*p* > 0.05	***p *= 0.0002**	*p* > 0.05	

^a,b,c^ Different initials indicate significant differences (*p* < 0.05) between events (T_basal_**_−_**, T1st_fear_, T1st_recovery_, T_basal__+_, T2nd_fear_, T2nd_recovery_). ^1,2^ Different numerals indicate significant differences (*p* < 0.05) between treatments (CONTROL,CBD, GABA, and SFP). Bold *p*-values represent statistically significant differences between events and treatments. Abbreviations of treatments: CONTROL—placebo; CBD—cannabidiol; GABA—gabapentin; SFP—synthetic facial pheromone. Abbreviations of evaluation times: T_basal_**_−_**—basal; T1st_fear_—first negative dog–cat interaction; T1st_recovery_—first rest; T_basal+_—neutral after drug administration; T2nd_fear_—second negative dog–cat interaction; T2nd_recovery_—second rest.

## Data Availability

Data are contained within the article.

## Supplementary Materials

The following supporting information can be downloaded at: https://www.mdpi.com/article/10.3390/vetsci12060523/s1, Supplementary Table S1: Correlation matrix for CONTROL group (treated with placebo); Supplementary Table S2: Correlation matrix for CBD group (treated with cannabidiol); Supplementary Table S3: Correlation matrix for GABA group (treated with gabapentin); Supplementary Table S4: Correlation matrix for SFP group (treated with synthetic facial pheromones).
