# Supplementary material for: Infrared Thermography and Physiological Variables as Methods for Recognizing Fear in Domestic Cats (Felis catus) Using Three Pharmacological Models: Cannabidiol, Gabapentin, and Synthetic Facial Pheromones
Source: Vet Sci. 2025 May 27;12(6):523. doi: 10.3390/vetsci12060523 (PMC12197366; doi:10.3390/vetsci12060523)
Supplement: Supplementary file 1 [file vetsci-12-00523-s001.zip › vetsci-3603830-supplementary.pdf]

Table S1. Correlation matrix for CONTROL group " Placebo"

	T°$_{REC}$	*HR*	*RR*	*T°$_{CAR}$*	*T°$_{OCU}$*	*T°$_{Upper\ eyeline}$*	*T°$_{Lower\ eyeline}$*	*T°$_{Rigth\ nostril}$*	*T°$_{Left\ nostril}$*	*T°$_{EAR}$*	*T°$_{Rigth\ whisk}$*	*T°$_{Left\ Whisk}$*	*T°$_{TLE}$*	*T°$_{FPL}$*	*T°$_{TLBB}$*	*T°$_{CHEST}$*	*T°$_{lumbar}$*
**T°$_{REC}$**	1																
**HR**	0.978	1															
	P< 0.001																
**RR**	0.983	0.956	1														
	P< 0.001	*P< 0.001*															
**T°$_{CAR}$**	0.988	0.977	0.978	1													
	P< 0.001	*P< 0.001*	*P< 0.001*														
**T°$_{OCU}$**	0.981	0.957	0.980	0.984	1												
	P< 0.001	*P< 0.001*	*P< 0.001*	*P< 0.001*													
**T°$_{Upper\ eyeline}$**	0.991	0.991	0.973	0.989	0.977	1											
	P< 0.001	*P< 0.001*	*P< 0.001*	*P< 0.001*	*P< 0.001*												
**T°$_{Lower\ eyeline}$**	0.832	0.766	0.835	0.793	0.821	0.781	1										
	P< 0.001	*P< 0.001*	*P< 0.001*	*P< 0.001*	*P< 0.001*	*P< 0.001*											
**T°$_{Rigth\ nostril}$**	0.992	0.977	0.983	0.995	0.987	0.990	0.821	1									
	P< 0.001	*P< 0.001*	*P< 0.001*	*P< 0.001*	*P< 0.001*	*P< 0.001*	*P< 0.001*										
**T°$_{Left\ nostril}$**	0.995	0.982	0.985	0.991	0.982	0.993	0.817	0.997	1								
	P< 0.001	*P< 0.001*	*P< 0.001*	*P< 0.001*	*P< 0.001*	*P< 0.001*	*P< 0.001*	*P< 0.001*									
**T°$_{EAR}$**	0.991	0.966	0.992	0.988	0.985	0.981	0.825	0.991	0.993	1							
	P< 0.001	*P< 0.001*	*P< 0.001*	*P< 0.001*	*P< 0.001*	*P< 0.001*	*P< 0.001*	*P< 0.001*	*P< 0.001*								
**T°$_{Rigth\ Whisk}$**	0.994	0.978	0.986	0.992	0.990	0.990	0.819	0.997	0.996	0.992	1						
	P< 0.001	*P< 0.001*	*P< 0.001*	*P< 0.001*	*P< 0.001*	*P< 0.001*	*P< 0.001*	*P< 0.001*	*P< 0.001*	*P< 0.001*							
**T°$_{Left\ Whisk}$**	0.987	0.973	0.975	0.993	0.980	0.988	0.774	0.994	0.993	0.985	0.991	1					
	P< 0.001	*P< 0.001*	*P< 0.001*	*P< 0.001*	*P< 0.001*	*P< 0.001*	*P< 0.001*	*P< 0.001*	*P< 0.001*	*P< 0.001*	*P< 0.001*						
**T°$_{TLE}$**	0.991	0.990	0.978	0.992	0.975	0.995	0.788	0.992	0.994	0.985	0.993	0.988	1				
	P< 0.001	*P< 0.001*	*P< 0.001*	*P< 0.001*	*P< 0.001*	*P< 0.001*	*P< 0.001*	*P< 0.001*	*P< 0.001*	*P< 0.001*	*P< 0.001*	*P< 0.001*					
**T°$_{FPL}$**	0.982	0.954	0.988	0.971	0.978	0.971	0.852	0.980	0.981	0.986	0.985	0.961	0.976	1			
	P< 0.001	*P< 0.001*	*P< 0.001*	*P< 0.001*	*P< 0.001*	*P< 0.001*	*P< 0.001*	*P< 0.001*	*P< 0.001*	*P< 0.001*	*P< 0.001*	*P< 0.001*	*P< 0.001*				
**T°$_{TLBB}$**	0.974	0.950	0.984	0.973	0.981	0.972	0.798	0.976	0.973	0.977	0.981	0.966	0.973	0.984	1		
	P< 0.001	*P< 0.001*	*P< 0.001*	*P< 0.001*	*P< 0.001*	*P< 0.001*	*P< 0.001*	*P< 0.001*	*P< 0.001*	*P< 0.001*	*P< 0.001*	*P< 0.001*	*P< 0.001*	*P< 0.001*			
**T°$_{CHEST}$**	0.990	0.984	0.982	0.993	0.985	0.995	0.803	0.992	0.994	0.988	0.993	0.987	0.994	0.980	0.980	1	
	P< 0.001	*P< 0.001*	*P< 0.001*	*P< 0.001*	*P< 0.001*	*P< 0.001*	*P< 0.001*	*P< 0.001*	*P< 0.001*	*P< 0.001*	*P< 0.001*	*P< 0.001*	*P< 0.001*	*P< 0.001*	*P< 0.001*		
**T°$_{Lumbar}$**	0.984	0.982	0.976	0.987	0.981	0.991	0.783	0.987	0.988	0.982	0.990	0.980	0.989	0.985	0.983	0.993	1
	P< 0.001	*P< 0.001*	*P< 0.001*	*P< 0.001*	*P< 0.001*	*P< 0.001*	*P< 0.001*	*P< 0.001*	*P< 0.001*	*P< 0.001*	*P< 0.001*	*P< 0.001*	*P< 0.001*	*P< 0.001*	*P< 0.001*	*P< 0.001*	

TABLE S2. Corelation matrix for CBD group " Cannabidiol"

	T°$_{REC}$	*HR*	*RR*	*T°$_{CAR}$*	*T°$_{OCU}$*	*T°$_{Upper\ eyeline}$*	*T°$_{Lower\ eyeline}$*	*T°$_{Rigth\ nostril}$*	*T°$_{Left\ nostril}$*	*T°$_{EAR}$*	*T°$_{Rigth\ whisk}$*	*T°$_{Left\ Whisk}$*	*T°$_{TLE}$*	*T°$_{FPL}$*	*T°$_{TLBB}$*	*T°$_{CHEST}$*	*T°$_{lumbar}$*
**T°$_{REC}$**	1																
**HR**	0.953	1															
	P< 0.001																
**RR**	0.984	0.962	1														
	P< 0.001	*P< 0.001*															
**T°$_{CAR}$**	0.979	0.911	0.970	1													
	P< 0.001	*P< 0.001*	*P< 0.001*														
**T°$_{OCU}$**	0.980	0.911	0.965	0.993	1												
	P< 0.001	*P< 0.001*	*P< 0.001*	*P< 0.001*													
**T°$_{Upper\ eyeline}$**	0.971	0.889	0.950	0.979	0.969	1											
	P< 0.001	*P< 0.001*	*P< 0.001*	*P< 0.001*	*P< 0.001*												
**T°$_{Lower\ eyeline}$**	0.981	0.906	0.957	0.987	0.988	0.986	1										
	P< 0.001	*P< 0.001*	*P< 0.001*	*P< 0.001*	*P< 0.001*	*P< 0.001*											
**T°$_{Rigth\ nostril}$**	0.979	0.911	0.947	0.973	0.980	0.979	0.990	1									
	P< 0.001	*P< 0.001*	*P< 0.001*	*P< 0.001*	*P< 0.001*	*P< 0.001*	*P< 0.001*										
**T°$_{Left\ nostril}$**	0.982	0.937	0.951	0.960	0.965	0.973	0.980	0.991	1								
	P< 0.001	*P< 0.001*	*P< 0.001*	*P< 0.001*	*P< 0.001*	*P< 0.001*	*P< 0.001*	*P< 0.001*									
**T°$_{EAR}$**	0.985	0.912	0.960	0.986	0.990	0.982	0.995	0.995	0.984	1							
	P< 0.001	*P< 0.001*	*P< 0.001*	*P< 0.001*	*P< 0.001*	*P< 0.001*	*P< 0.001*	*P< 0.001*	*P< 0.001*								
**T°$_{Rigth\ Whisk}$**	0.981	0.928	0.951	0.962	0.977	0.960	0.980	0.991	0.990	0.987	1						
	P< 0.001	*P< 0.001*	*P< 0.001*	*P< 0.001*	*P< 0.001*	*P< 0.001*	*P< 0.001*	*P< 0.001*	*P< 0.001*	*P< 0.001*							
**T°$_{Left\ Whisk}$**	0.983	0.926	0.959	0.975	0.985	0.968	0.989	0.994	0.987	0.994	0.993	1					
	P< 0.001	*P< 0.001*	*P< 0.001*	*P< 0.001*	*P< 0.001*	*P< 0.001*	*P< 0.001*	*P< 0.001*	*P< 0.001*	*P< 0.001*	*P< 0.001*						
**T°$_{TLE}$**	0.969	0.893	0.947	0.990	0.989	0.976	0.986	0.982	0.966	0.989	0.968	0.980	1				
	P< 0.001	*P< 0.001*	*P< 0.001*	*P< 0.001*	*P< 0.001*	*P< 0.001*	*P< 0.001*	*P< 0.001*	*P< 0.001*	*P< 0.001*	*P< 0.001*	*P< 0.001*					
**T°$_{FPL}$**	0.975	0.904	0.943	0.979	0.986	0.974	0.992	0.993	0.982	0.995	0.987	0.992	0.989	1			
	P< 0.001	*P< 0.001*	*P< 0.001*	*P< 0.001*	*P< 0.001*	*P< 0.001*	*P< 0.001*	*P< 0.001*	*P< 0.001*	*P< 0.001*	*P< 0.001*	*P< 0.001*	*P< 0.001*				
**T°$_{TLBB}$**	0.975	0.912	0.953	0.975	0.987	0.963	0.987	0.991	0.978	0.992	0.991	0.996	0.981	0.990	1		
	P< 0.001	*P< 0.001*	*P< 0.001*	*P< 0.001*	*P< 0.001*	*P< 0.001*	*P< 0.001*	*P< 0.001*	*P< 0.001*	*P< 0.001*	*P< 0.001*	*P< 0.001*	*P< 0.001*	*P< 0.001*			
**T°$_{CHEST}$**	0.984	0.911	0.957	0.981	0.984	0.986	0.995	0.997	0.989	0.997	0.990	0.993	0.985	0.993	0.990	1	
	P< 0.001	*P< 0.001*	*P< 0.001*	*P< 0.001*	*P< 0.001*	*P< 0.001*	*P< 0.001*	*P< 0.001*	*P< 0.001*	*P< 0.001*	*P< 0.001*	*P< 0.001*	*P< 0.001*	*P< 0.001*	*P< 0.001*		
**T°$_{Lumbar}$**	0.988	0.943	0.961	0.970	0.978	0.968	0.986	0.990	0.993	0.989	0.994	0.992	0.970	0.987	0.985	0.990	1
	P< 0.001	*P< 0.001*	*P< 0.001*	*P< 0.001*	*P< 0.001*	*P< 0.001*	*P< 0.001*	*P< 0.001*	*P< 0.001*	*P< 0.001*	*P< 0.001*	*P< 0.001*	*P< 0.001*	*P< 0.001*	*P< 0.001*	*P< 0.001*	

TABLE S3. Corelation matrix for GABA group " Gabapentine"

	T° $_{REC}$	*HR*	*RR*	*T° $_{CAR}$*	*T° $_{OCU}$*	*T° $_{Upper\ eyeline}$*	*T° $_{Lower\ eyeline}$*	*T° $_{Rigth\ nostril}$*	*T° $_{Left\ nostril}$*	*T° $_{EAR}$*	*T° $_{Rigth\ whisk}$*	*T° $_{Left\ Whisk}$*	*T° $_{TLE}$*	*T° $_{FPL}$*	*T° $_{TLBB}$*	*T° $_{CHEST}$*	*T° $_{lumbar}$*
**T°$_{REC}$**	1																
**HR**	0.929	1															
	P< 0.001																
**RR**	0.984	0.970	1														
	P< 0.001	*P< 0.001*															
**T°$_{CAR}$**	0.992	0.941	0.985	1													
	P< 0.001	*P< 0.001*	*P< 0.001*														
**T°$_{OCU}$**	0.986	0.938	0.983	0.988	1												
	P< 0.001	*P< 0.001*	*P< 0.001*	*P< 0.001*													
**T°$_{Upper\ eyeline}$**	0.971	0.916	0.964	0.975	0.987	1											
	P< 0.001	*P< 0.001*	*P< 0.001*	*P< 0.001*	*P< 0.001*												
**T°$_{Lower\ eyeline}$**	0.985	0.923	0.978	0.984	0.992	0.988	1										
	P< 0.001	*P< 0.001*	*P< 0.001*	*P< 0.001*	*P< 0.001*	*P< 0.001*											
**T°$_{Rigth\ nostril}$**	0.983	0.875	0.949	0.981	0.972	0.961	0.972	1									
	P< 0.001	*P< 0.001*	*P< 0.001*	*P< 0.001*	*P< 0.001*	*P< 0.001*	*P< 0.001*										
**T°$_{Left\ nostril}$**	0.989	0.878	0.957	0.976	0.974	0.963	0.975	0.989	1								
	P< 0.001	*P< 0.001*	*P< 0.001*	*P< 0.001*	*P< 0.001*	*P< 0.001*	*P< 0.001*	*P< 0.001*									
**T°$_{EAR}$**	0.988	0.883	0.958	0.985	0.976	0.966	0.978	0.996	0.993	1							
	P< 0.001	*P< 0.001*	*P< 0.001*	*P< 0.001*	*P< 0.001*	*P< 0.001*	*P< 0.001*	*P< 0.001*	*P< 0.001*								
**T°$_{Rigth\ Whisk}$**	0.976	0.851	0.935	0.970	0.962	0.950	0.963	0.992	0.989	0.994	1						
	P< 0.001	*P< 0.001*	*P< 0.001*	*P< 0.001*	*P< 0.001*	*P< 0.001*	*P< 0.001*	*P< 0.001*	*P< 0.001*	*P< 0.001*							
**T°$_{Left\ Whisk}$**	0.986	0.884	0.955	0.980	0.972	0.961	0.972	0.989	0.991	0.994	0.993	1					
	P< 0.001	*P< 0.001*	*P< 0.001*	*P< 0.001*	*P< 0.001*	*P< 0.001*	*P< 0.001*	*P< 0.001*	*P< 0.001*	*P< 0.001*	*P< 0.001*						
**T°$_{TLE}$**	0.989	0.919	0.976	0.989	0.986	0.974	0.990	0.985	0.984	0.990	0.982	0.990	1				
	P< 0.001	*P< 0.001*	*P< 0.001*	*P< 0.001*	*P< 0.001*	*P< 0.001*	*P< 0.001*	*P< 0.001*	*P< 0.001*	*P< 0.001*	*P< 0.001*	*P< 0.001*					
**T°$_{FPL}$**	0.963	0.830	0.925	0.953	0.958	0.966	0.972	0.971	0.981	0.977	0.979	0.976	0.965	1			
	P< 0.001	*P< 0.001*	*P< 0.001*	*P< 0.001*	*P< 0.001*	*P< 0.001*	*P< 0.001*	*P< 0.001*	*P< 0.001*	*P< 0.001*	*P< 0.001*	*P< 0.001*	*P< 0.001*				
**T°$_{TLBB}$**	0.987	0.894	0.961	0.978	0.976	0.961	0.974	0.986	0.990	0.991	0.990	0.993	0.992	0.967	1		
	P< 0.001	*P< 0.001*	*P< 0.001*	*P< 0.001*	*P< 0.001*	*P< 0.001*	*P< 0.001*	*P< 0.001*	*P< 0.001*	*P< 0.001*	*P< 0.001*	*P< 0.001*	*P< 0.001*	*P< 0.001*			
**T°$_{CHEST}$**	0.985	0.899	0.962	0.989	0.981	0.975	0.980	0.993	0.982	0.993	0.985	0.986	0.988	0.966	0.982	1	
	P< 0.001	*P< 0.001*	*P< 0.001*	*P< 0.001*	*P< 0.001*	*P< 0.001*	*P< 0.001*	*P< 0.001*	*P< 0.001*	*P< 0.001*	*P< 0.001*	*P< 0.001*	*P< 0.001*	*P< 0.001*	*P< 0.001*		
**T°$_{Lumbar}$**	0.972	0.904	0.960	0.981	0.981	0.978	0.984	0.971	0.964	0.975	0.973	0.976	0.985	0.966	0.974	0.983	1
	P< 0.001	*P< 0.001*	*P< 0.001*	*P< 0.001*	*P< 0.001*	*P< 0.001*	*P< 0.001*	*P< 0.001*	*P< 0.001*	*P< 0.001*	*P< 0.001*	*P< 0.001*	*P< 0.001*	*P< 0.001*	*P< 0.001*	*P< 0.001*	

TABLE S4. Corelation matrix for SFP group " Synthetic Facial Pheromones"

	T° $_{REC}$	*HR*	*RR*	*T° $_{CAR}$*	*T° $_{OCU}$*	*T° $_{Upper\ eyeline}$*	*T° $_{Lower\ eyeline}$*	*T° $_{Rigth\ nostril}$*	*T° $_{Left\ nostril}$*	*T° $_{EAR}$*	*T° $_{Rigth\ whisk}$*	*T° $_{Left\ Whisk}$*	*T° $_{TLE}$*	*T° $_{FPL}$*	*T° $_{TLBB}$*	*T° $_{CHEST}$*	*T° $_{lumbar}$*
**T°$_{REC}$**	1																
**HR**	0.900	1															
	P< 0.001																
**RR**	0.927	0.982	1														
	P< 0.001	*P< 0.001*															
**T°$_{CAR}$**	0.980	0.950	0.972	1													
	P< 0.001	*P< 0.001*	*P< 0.001*														
**T°$_{OCU}$**	0.968	0.972	0.976	0.993	1												
	P< 0.001	*P< 0.001*	*P< 0.001*	*P< 0.001*													
**T°$_{Upper\ eyeline}$**	0.970	0.969	0.982	0.990	0.990	1											
	P< 0.001	*P< 0.001*	*P< 0.001*	*P< 0.001*	*P< 0.001*												
**T°$_{Lower\ eyeline}$**	0.981	0.939	0.962	0.991	0.981	0.984	1										
	P< 0.001	*P< 0.001*	*P< 0.001*	*P< 0.001*	*P< 0.001*	*P< 0.001*											
**T°$_{Rigth\ nostril}$**	0.962	0.944	0.973	0.988	0.983	0.982	0.980	1									
	P< 0.001	*P< 0.001*	*P< 0.001*	*P< 0.001*	*P< 0.001*	*P< 0.001*	*P< 0.001*										
**T°$_{Left\ nostril}$**	0.967	0.948	0.974	0.992	0.987	0.984	0.985	0.996	1								
	P< 0.001	*P< 0.001*	*P< 0.001*	*P< 0.001*	*P< 0.001*	*P< 0.001*	*P< 0.001*	*P< 0.001*									
**T°$_{EAR}$**	0.971	0.830	0.879	0.944	0.917	0.927	0.961	0.928	0.935	1							
	P< 0.001	*P< 0.001*	*P< 0.001*	*P< 0.001*	*P< 0.001*	*P< 0.001*	*P< 0.001*	*P< 0.001*	*P< 0.001*								
**T°$_{Rigth\ Whisk}$**	0.976	0.962	0.977	0.993	0.992	0.992	0.984	0.986	0.989	0.929	1						
	P< 0.001	*P< 0.001*	*P< 0.001*	*P< 0.001*	*P< 0.001*	*P< 0.001*	*P< 0.001*	*P< 0.001*	*P< 0.001*	*P< 0.001*	*P< 0.001*						
**T°$_{Left\ Whisk}$**	0.977	0.956	0.973	0.993	0.992	0.991	0.983	0.988	0.992	0.932	0.997	1					
	P< 0.001	*P< 0.001*	*P< 0.001*	*P< 0.001*	*P< 0.001*	*P< 0.001*	*P< 0.001*	*P< 0.001*	*P< 0.001*	*P< 0.001*	*P< 0.001*						
**T°$_{TLE}$**	0.725	0.613	0.671	0.721	0.663	0.693	0.766	0.682	0.701	0.834	0.676	0.671	1				
	P< 0.001	*P< 0.001*	*P< 0.001*	*P< 0.001*	*P< 0.001*	*P< 0.001*	*P< 0.001*	*P< 0.001*	*P< 0.001*	*P< 0.001*	*P< 0.001*	*P< 0.001*					
**T°$_{FPL}$**	0.980	0.895	0.933	0.974	0.959	0.967	0.978	0.979	0.980	0.969	0.972	0.977	0.718	1			
	P< 0.001	*P< 0.001*	*P< 0.001*	*P< 0.001*	*P< 0.001*	*P< 0.001*	*P< 0.001*	*P< 0.001*	*P< 0.001*	*P< 0.001*	*P< 0.001*	*P< 0.001*	*P< 0.001*				
**T°$_{TLBB}$**	0.986	0.930	0.953	0.985	0.979	0.983	0.984	0.982	0.986	0.956	0.989	0.990	0.701	0.989	1		
	P< 0.001	*P< 0.001*	*P< 0.001*	*P< 0.001*	*P< 0.001*	*P< 0.001*	*P< 0.001*	*P< 0.001*	*P< 0.001*	*P< 0.001*	*P< 0.001*	*P< 0.001*	*P< 0.001*	*P< 0.001*			
**T°$_{CHEST}$**	0.986	0.897	0.932	0.975	0.962	0.967	0.979	0.969	0.972	0.978	0.974	0.976	0.727	0.991	0.986	1	
	P< 0.001	*P< 0.001*	*P< 0.001*	*P< 0.001*	*P< 0.001*	*P< 0.001*	*P< 0.001*	*P< 0.001*	*P< 0.001*	*P< 0.001*	*P< 0.001*	*P< 0.001*	*P< 0.001*	*P< 0.001*	*P< 0.001*		
**T°$_{Lumbar}$**	0.982	0.905	0.938	0.975	0.960	0.971	0.980	0.975	0.975	0.974	0.976	0.977	0.719	0.994	0.987	0.996	1
	P< 0.001	*P< 0.001*	*P< 0.001*	*P< 0.001*	*P< 0.001*	*P< 0.001*	*P< 0.001*	*P< 0.001*	*P< 0.001*	*P< 0.001*	*P< 0.001*	*P< 0.001*	*P< 0.001*	*P< 0.001*	*P< 0.001*	*P< 0.001*	
